# Selectively Enhanced UV-A Photoresponsivity of a GaN MSM UV Photodetector with a Step-Graded Al_x_Ga_1−x_N Buffer Layer

**DOI:** 10.3390/s17071684

**Published:** 2017-07-21

**Authors:** Chang-Ju Lee, Chul-Ho Won, Jung-Hee Lee, Sung-Ho Hahm, Hongsik Park

**Affiliations:** School of Electronics Engineering, College of IT Engineering, Kyungpook National University, Daegu 41566, Korea; chjlee@knu.ac.kr (C.-J.L.); chwon@ee.knu.ac.kr (C.-H.W.); jlee@ee.knu.ac.kr (J.-H.L.); shhahm@knu.ac.kr (S.-H.H.)

**Keywords:** gallium nitride (GaN), ultraviolet (UV), photodetector, UV-to-visible rejection ratio, step-graded Al_x_Ga_1−x_N buffer layer

## Abstract

The UV-to-visible rejection ratio is one of the important figure of merits of GaN-based UV photodetectors. For cost-effectiveness and large-scale fabrication of GaN devices, we tried to grow a GaN epitaxial layer on silicon substrate with complicated buffer layers for a stress-release. It is known that the structure of the buffer layers affects the performance of devices fabricated on the GaN epitaxial layers. In this study, we show that the design of a buffer layer structure can make effect on the UV-to-visible rejection ratio of GaN UV photodetectors. The GaN photodetector fabricated on GaN-on-silicon substrate with a step-graded Al_x_Ga_−x_N buffer layer has a highly-selective photoresponse at 365-nm wavelength. The UV-to-visible rejection ratio of the GaN UV photodetector with the step-graded Al_x_Ga_1−x_N buffer layer was an order-of-magnitude higher than that of a photodetector with a conventional GaN/AlN multi buffer layer. The maximum photoresponsivity was as high as 5 × 10^−2^ A/W. This result implies that the design of buffer layer is important for photoresponse characteristics of GaN UV photodetectors as well as the crystal quality of the GaN epitaxial layers.

## 1. Introduction

GaN-based UV photodetectors have been actively studied for various commercial applications such as ozone layer monitoring systems, flame sensors, and medical inspection systems [1,2,3]. For the last two decades, many studies have examined the GaN-based UV photodetectors with different device structures such as p-i-n, Schottky, and metal-semiconductor-metal (MSM) types [4,5,6,7,8,9,10,11,12,13,14,15,16]. Among them, the MSM-type UV photodetectors have the advantages of a low dark current level back-to-back diode structure and a high process compatibility with other electronic devices in the circuit level design due to their simple fabrication process [9,10,11,12,13,14,15,16].

Recently, silicon wafers have been used as substrates for growing GaN epitaxial layers to reduce process cost and to enable large-scale fabrication of GaN-based devices [17,18]. However, GaN epitaxial layers grown on silicon substrates typically have a strong tensile stress and a high density of defects which limit the performance of GaN photodetector such as the UV-to-visible rejection ratio and responsivity [19]. Recent studies on the growth of GaN on silicon report that an AlGaN buffer layer with a gradually-changing Al mole fraction can effectively suppress the tensile stress and improve the quality of epitaxial GaN layers [20,21]. AlGaN-based buffer structures have been widely used for GaN or AlGaN layer growth on various substrates such as silicon, sapphire, and bulk GaN, and these epitaxial layer structures were applied for fabrication of various types of GaN photodetectors like AlGaN-based p-i-n UV photodetector [22], Al_x_Ga_1−x_N high-gain avalanche UV photodetectors [23,24], AlGaN/GaN UV photodetectors [25,26], and quadruple-band Al_x_Ga_1−x_N UV photodetector [27]. The improvement of the quality of epitaxial layers by using the step-graded AlGaN buffer structures resulted in the improved device performances. 

In this study, we investigated the effects of buffer layers on the photoresponse characteristics of GaN MSM UV photodetectors. We fabricated and evaluated MSM photodetectors on GaN grown on silicon substrate with a step-graded Al_x_Ga_1−x_N. To study the effect of the buffer layer, we also fabricated a GaN MSM UV photodetector using the same fabrication process and GaN epitaxial layer with a conventional buffer layer [high-temperature (HT)-GaN/low temperature (LT) AlN multi-layer] and compared the photoresponse characteristics of the photodetectors. The GaN MSM UV photodetector with the step-graded Al_x_Ga_1−x_N buffer layer had a higher photoresponsivity at the wavelength range near 365 nm (UV-A region).

## 2. Materials and Methods

Two types of GaN epitaxial layers with different buffer layers were grown on n-type silicon(111) substrates by metal organic chemical vapor deposition (MOCVD). The trimethylgallium (TMGa), trimethylaluminum (TMAl), and ammonia (NH_3_) were used as precursors for Ga, Al, N, respectively. Silicon substrates were cleaned by diluted hydrogen fluoride (HF, 1%) to remove the native oxide on the silicon surface. For the growth of the first-type GaN epitaxial layer, a 100-nm-thick HT-AlN buffer layer was grown on silicon at 1100 °C to prevent a melt-back etching effect and to release the strong tensile stress between the GaN epitaxial layer and silicon substrate. 

**Figure 1:**
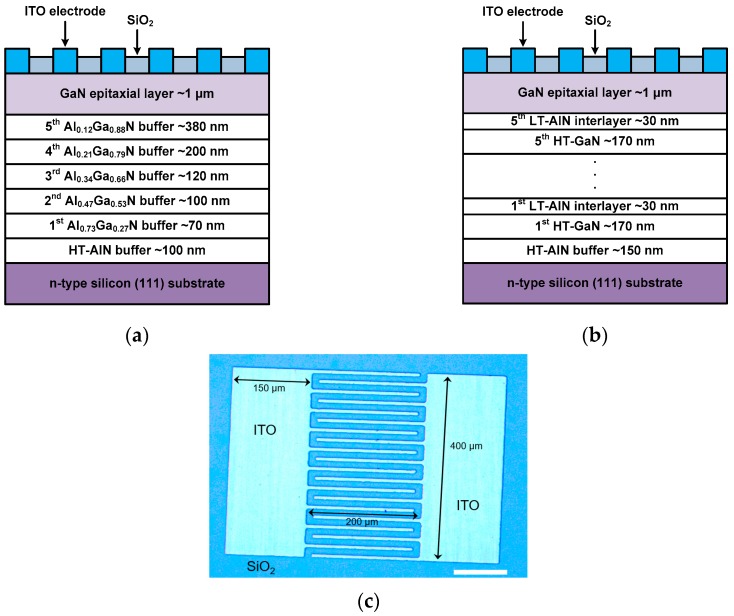
Schematic cross-sectional views of GaN metal-semiconductor-metal (MSM) UV photodetectors showing epitaxial layer structures with different buffer layers and top view image of the fabricated UV photodetector. (**a**) Epitaxial layer structure with a step-graded Al_x_Ga_1−x_N buffer layer and (**b**) epitaxial layer structure with a conventionally-used high-temperature(HT)-GaN/low-temperature(LT)-AlN buffer layer; (**c**) Optical image of the GaN MSM UV photodetector. The design of Schottky electrode structure was identical for both types of devices. The thickness of the GaN active layer, material for the Schottky contact, and device fabrication process for both devices were also the same.

After the HT-AlN growth, a step-graded Al_x_Ga_1−x_N buffer layer that is consisted of five stacks of Al_x_Ga_1−x_N layers with gradual Al compositions were grown on the HT-AlN buffer layer (whole epitaxial layer structure is shown in Figure 1a). For the growth of the second-type GaN epitaxial layer, a 150-nm-thick HT-AlN buffer layer was grown on silicon at 1100 °C, followed by five stacks of a HT-GaN (170-nm-thick)/LT-AlN (30-nm-thick) multi-layer were grown on the HT-AlN buffer layer (whole epitaxial layer structure is shown in Figure 1b). After the growth of the buffer layer, a GaN active layer with the thickness of 1 μm was grown on each type of buffer layer. The growth temperature (1070 °C) and growth pressure (100 Torr) for the GaN active layer growth were identical for the two types of epitaxial structures. Since MSM-type UV photodetectors require a very low dark current and a good Schottky contact, we grew highly resistive GaN active layers without any doping. The polarity of the GaN active layer is typically n-type. The resistivity of the undoped GaN active layer is extremely high so that a good ohmic contact formation for conductivity characterization is very difficult. After the epitaxial growth of GaN, an SiO_2_ (thickness of 110 nm) for surface passivation was deposited by plasma-enhanced chemical vapor deposition (PECVD) at 300 °C. After the deposition of the passivation layer, Schottky electrodes were patterned patterns through photolithography, etching of the passivation layer on the electrode regions, the deposition of 100-nm-thick indium-tin-oxide (ITO), and lift-off process. The ITO layer for the Schottky electrodes was deposited by a radio-frequency magnetron sputtering system; the working pressure was 10 mTorr in an Ar/O_2_ atmosphere (Ar:O_2_ = 1000:1), and the deposition temperature was 300 °C.

Figure 1c shows the top view image of the GaN MSM UV photodetector. The area of the device region of the fabricated UV photodetectors was 400 μm × 500 μm with finger-type electrodes (figure length: 200 μm, finger width: 10 μm, and the gap between figure electrodes: 10 μm). The electrical properties of the fabricated GaN MSM UV photodetectors were characterized by using a semiconductor parameter analyzer (Agilent 4156C). The photoresponsive I-V characteristics and spectral photoresponsivity were characterized by using a 150 W Xenon arc lamp, a monochromator system (Oriel 74000), a low power detector (Newport 918), and a power meter (Newport 1930C).

## 3. Results and Discussion

**Figure 2:**
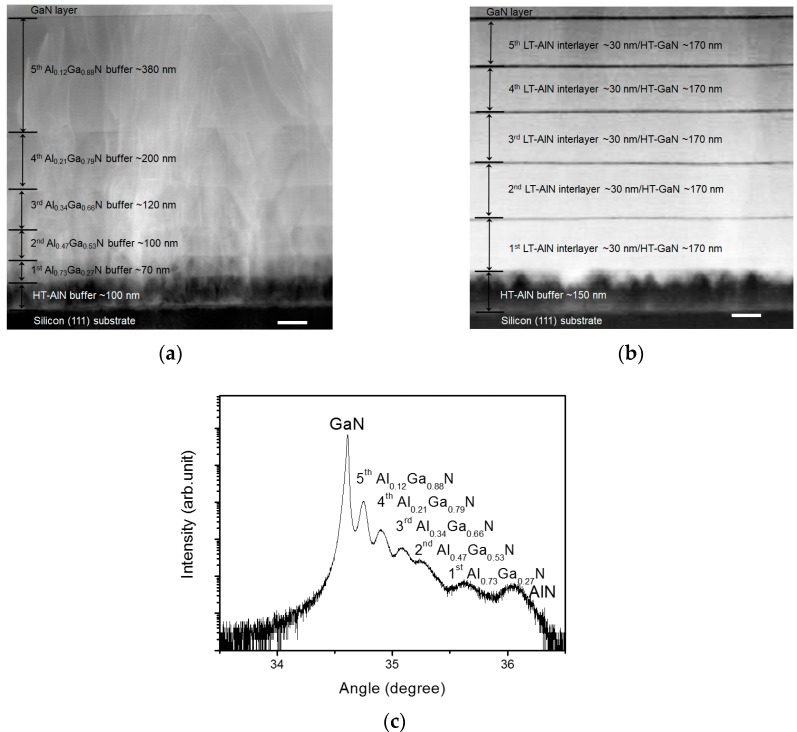
Transmission electron microscope (TEM) images of GaN epitaxial layer structures with different buffer layers. (**a**) Epitaxial layer structure with a step-graded Al_x_Ga_1−x_N buffer layer and (**b**) epitaxial layer structure with a conventionally-used HT-GaN/LT-AlN buffer layer (scale bar: 100 nm); (**c**) high-resolution X-ray diffraction (HR-XRD) 2θ−ω scan of the GaN epitaxial layer grown on silicon substrate with the step-graded Al_x_Ga_1−x_N buffer layer, showing the Al mole fractions of the buffer structure.

Figure 2a shows a transmission electron microscope (TEM) image of the cross-section of the GaN epitaxial layer grown on silicon(111) substrate with the step-graded Al_x_Ga_1−x_N buffer layer. The thicknesses of each Al_x_Ga_1−x_N layer measured from the TEM image were 70 (the bottom layer of the buffer structure), 100, 120, 200, and 380 nm (the top layer of the buffer structure), respectively. It is observed that each layer in the buffer structure is thicker than the underneath layer. The reason for this increased thickness is that we fixed the growth time for all Al_x_Ga_1−x_N layers and the growth rate for Al_x_Ga_1−x_N with a smaller Al composition is typically higher than that for Al_x_Ga_1−x_N with a larger Al composition. Figure 2b shows a TEM image of the cross-section of the GaN epitaxial layer grown on silicon(111) substrate with the conventionally-used HT-GaN/LT-AlN buffer layer. The thicknesses of each HT-GaN and LT-AlN layer measured from the TEM image were 170 and 30 nm, respectively. Figure 2c shows the 2θ−ω scan spectrum of a high-resolution X-ray diffraction (HR-XRD) measurement on the GaN epitaxial layer with the step-graded Al_x_Ga_1−x_N buffer layer. Each peak indicates the GaN, Al_x_Ga_1−x_N, and AlN layers. The intensity of the peaks in XRD 2θ−ω scan measurement typically depends on the crystal quality and thickness of a measured crystal. Figure 2c indicates that the crystal quality of the Al_x_Ga_1−x_N layers of the buffer was improved and the layer thickness was increased as the Al mole fraction of the Al_x_Ga_1−x_N was reduced. From the XRD scan, we can estimate the Al composition of Al_x_Ga_1−x_N layers by Vegard’s law [28],

(1) $$x=\left(\frac{a_{GaN}}{a_{AlN}-a_{GaN}}\right)\cdot \varepsilon_{rel}$$

where $a_{GaN}$ and $a_{AlN}$ are the lattice constant of GaN and AlN and $\varepsilon_{rel}$ is the fully relaxed strain of GaN on AlN. Assuming the AlGaN layer is fully strained, $\varepsilon_{rel}$ is calculated from the perpendicular strain rate and a correction factor as given by

(2) $$\varepsilon_{rel}=C\;\cdot \;\varepsilon^{⊥}$$

where, C is a correction factor and $\varepsilon^{⊥}$ is a perpendicular strain rate that is calculated from the peak positions in the XRD measurement. The Al composition of each Al_x_Ga_1−x_N layer calculated from the Equation (1) were 73 (the bottom layer of the buffer structure), 47, 34, 21, and 12% (the top layer of the buffer structure), respectively. As reported in literature, the step-graded Al_x_Ga_1−x_N structure is an effective buffer layer for the growth of GaN-on-silicon with reduced dislocation density. The incremental increase of Al mole fraction in the Al_x_Ga_1−x_N multi-layer significantly reduce the effect of lattice mismatch between GaN and silicon, which results in a reduced density of defects in the GaN layers due to effectively terminated propagation of dislocations [29,30].

**Figure 3:**
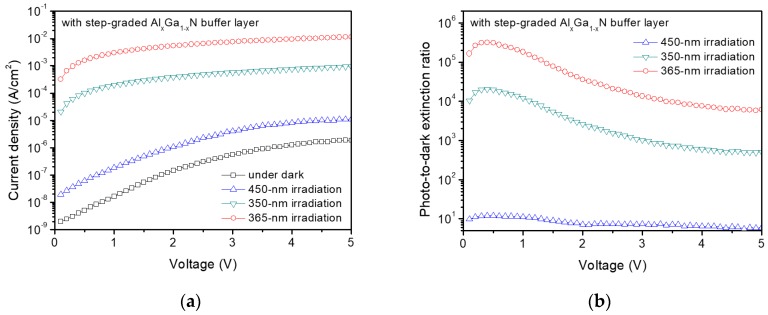
Electrical characteristics of GaN MSM UV photodetector with the step-graded Al_x_Ga_1−x_N buffer layer. (**a**) Current-voltage (I-V) characteristics and (**b**) photo-to-dark extinction ratios of the GaN photodetector at dark condition and under UV illumination with different wavelengths.

Figure 3a shows dark and photoresponsive current-voltage (I-V) characteristics of the GaN MSM UV photodetector with the step-graded Al_x_Ga_1−x_N buffer layer under dark condition and irradiation of light with wavelengths of 350 nm, 365 nm, and 450 nm. The photocurrent under UV irradiation is about 10^3^~10^4^ times higher than that under the irradiation of visible light. The dark current density was as low as 1.6 × 10^−8^ A/cm^2^ at 1 V bias, which means that the Schottky barrier can effectively block carriers at a low-field condition. The plot shows that the detector is also weakly response to visible light. The current density with under 450-nm UV illumination is one order of magnitude higher than the dark current density. The response of the GaN detector to light with an energy lower than the GaN band gap can be explained by light absorption through defects in the GaN epitaxial layer and interface traps in the ITO/GaN Schottky junctions. However, the photocurrent under the visible light is much lower than that under UV light. This indicates that the crystal quality of the GaN epitaxial layer and the interface of ITO/GaN junctions are sufficiently good and suitable for the fabrication of UV photodetectors. It is also notable that the current density under 365-nm UV irradiation was 3.0 × 10^−3^ A/cm^2^ at 1 V bias which is an order-of-magnitude higher than the current density under 350-nm UV irradiation. Considering the irradiation power density of 350-nm and 365-nm UV for the measurement was almost the same, the responsivity of the detector under the 365-nm UV irradiation is about 10 times higher than that under the 350-nm UV irradiation. This wavelength dependent UV response will be discussed with the result of the spectral responsivity measurement below. The photo-to-dark extinction ratio of the GaN UV photodetector under 365-nm UV irradiation exceeded 10^5^ until 1 V bias as shown in Figure 3b. It is observed that the photo-to-dark extinction ratio is gradually decreased at the bias larger than 1 V bias due to the increase of the dark current density. The maximum photo-to-dark extinction ratio is observed at the low bias range. The reason for this bias-dependent photo-to-dark extinction ratio can be explained by the fact that the dark current is significantly dependent on the bias voltage while the photocurrent is not directly dependent on the bias voltage. The photocurrent level is dominantly dependent on the number of photo-generated carriers not on the electric field in the GaN channel but the dark current that is contributed from the surface or the Schottky junction leakage current can be significantly affected by the bias voltage.

**Figure 4:**
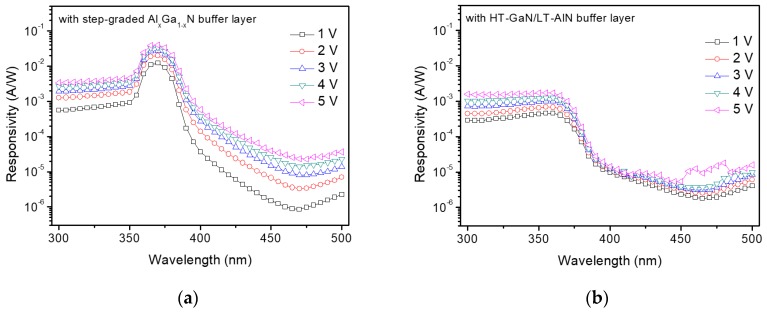
Spectral photoresponsivity characteristics of GaN MSM UV photodetectors with (**a**) the step-graded Al_x_Ga_1−x_N buffer layer and (**b**) HT-GaN/LT-AlN buffer layer, indicating significantly increased photoresponsivity of the GaN MSM UV photodetector with the step-graded Al_x_Ga_1−x_N buffer layer near 365-nm wavelength region (UV-A region).

To investigate the effects of the buffer layers on the performance of GaN MSM UV photodetectors, we compared the photoresponsive characteristics of the two types of GaN MSM UV photodetectors that were fabricated with the GaN epitaxial layers grown on the different buffer structures. Figure 4a shows the spectral photoresponsivity characteristics of the GaN MSM UV photodetector with the step-graded Al_x_Ga_1−x_N buffer layer (the first-type) and Figure 4b shows that of the GaN MSM UV photodetector with the HT-GaN/LT-AlN buffer layer (the second-type). The maximum responsivity of the first-type photodetector was 5 × 10^−2^ A/W, whereas that of the second-type photodetector was 2 × 10^−3^ A/W. The clear difference between the characteristics of the two types of UV photodetector is the significantly enhanced responsivity of the first-type photodetector in the wavelength near 365 nm (UV-A region) which is the wavelength of UV light corresponding to the bandgap energy of GaN. The responsivity in the wavelength shorter than 350 nm was one order of magnitude lower than the maximum value. This wavelength-selective responsivity of the GaN MSM UV photodetector with the step-graded Al_x_Ga_1−x_N buffer layer is an unusual photoresponsive characteristic that has not reported in the literature yet. As shown in Figure 4b, the GaN MSM UV photodetector fabricated with the conventional buffer layer (HT-GaN/LT-AlN multi-layer) did not show a wavelength-selective photoresponse in the UV range. The possibility that the GaN UV photodetector with the step-graded Al_x_Ga_1−x_N buffer layer is also more sensitive to the visible light than the second-type device because of absorption of reflected photons with wavelengths larger than 365 nm. The slightly higher sensitivity of the first-type device in the visible wavelength range may be explained by increased number of absorbed photons.

**Figure 5:**
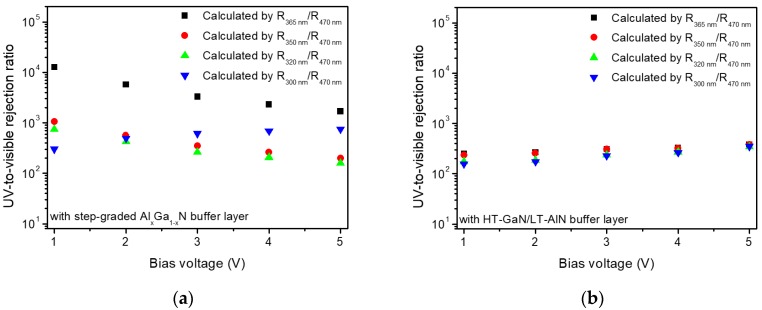
UV-to-visible rejection ratio of GaN MSM UV photodetectors with (**a**) the step-graded Al_x_Ga_1−x_N buffer layer and (**b**) HT-GaN/LT-AlN buffer layer, showing significantly enhanced UV-to-visible rejection ratio of the GaN MSM UV photodetector with the step-graded Al_x_Ga_1−x_N buffer layer at 365-nm wavelength.

Figure 5a,b show the UV-to-visible rejection ratio of the two types of GaN MSM UV which is one of important figure of merits of UV photodetectors. The UV-to-visible rejection ratios were calculated by using the responsivity values at UV (300, 320, 350, 365 nm) and visible (470 nm) wavelengths obtained from the Figure 4a,b.

It is clearly shown that the UV-to-visible rejection ratio of the first-type detector (step-graded Al_x_Ga_1−x_N buffer) is significantly enhanced at the specific wavelength range near 365 nm. The UV-to-visible rejection ratio at 365 nm (R_365-nm_/R_470-nm_) is almost one order of magnitude higher than that of UV wavelength range shorter than 365 nm. On the other hand, the second-type detector (HT-GaN/LT-AlN buffer) has relatively constant UV-to-visible rejection ratio in the UV range measured in this experiment as also shown in Figure 4b. This photoresponse characteristic of the two types of detectors implies that the selectively enhanced responsivity of the first-type detector comes from the difference in the buffer structures. Figure 5a,b show that the UV-to-visible rejection ratio is less dependent on the bias voltage compared to the photo-to-dark extinction ratio shown in Figure 3b. In the case of 365-nm wavelength, the maximum value of the UV-to-visible rejection ratio was observed in the low bias range, which is suitable for UV photodetector application typically requiring a low operating voltage.

**Figure 6:**
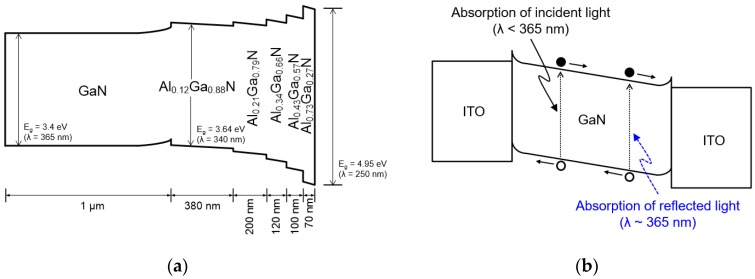
Energy band diagram of (**a**) the epitaxial structure of the GaN layer with the step-graded Al_x_Ga_1−x_N buffer layer; (**b**) GaN MSM UV photodetector under 365-nm UV irradiation with reflection and reabsorption effect of incident light.

The highly wavelength-selective photoresponse of the MSM UV photodetector with the graded Al_x_Ga_1−x_N buffer layer in the UV-A region can be explained by the enhancement of quantum efficiency caused from a multiple reflection of incident UV light at the interfaces of Al_x_Ga_1−x_N layers and absorption of the reflected light [31]. Figure 6a shows the energy band structure of the GaN and step-graded Al_x_Ga_1−x_N buffer layer with different Al compositions in which the polarization effect is considered. The energy bandgap of the Al_x_Ga_1−x_N layers gradually increases from the GaN layer to silicon substrate. Incident UV light with wavelength larger than 365 nm that corresponds to the bandgap of GaN is absorbed in the GaN layer first. The light that passed through the GaN layer is reflected at the interface between the bottom AlGaN layer (Al_0.73_Ga_0.27_N) and silicon substrate. The reflected light with wavelength smaller than 365 nm is dominantly absorbed through the Al_x_Ga_1−x_N layers and the light with wavelength larger than 365 nm, energy is smaller than the bandgap of GaN or AlGaN, does not significantly contribute to the photoresponsive current. In the case of the GaN MSM UV photodetector with HT-GaN/LT-AlN buffer, the incident UV light passing through the GaN layer does not contribute to the photocurrent again because the UV light is absorbed in the HT-GaN layers and defective LT-AlN layers in the buffer structure.

These results mean that the design of the buffer structures for the GaN on Si can affect the photoresponse characteristics as well as the quality of GaN epitaxial layers. The maximum responsivity of the first-type device is 2~3 times less than the best reported results [9,10,11,12,13,14,15,16] and the maximum UV-to-visible rejection ratio of the first-type device is comparable with the best results. To improve the responsivity of this type of device, it will be necessary to optimize device design and structure as well as the crystal quality of active epitaxial layers. The wavelength-selective enhancement of the GaN UV detector responsivity can be useful for UV sensors applied for flame detection or missile detection.

## 4. Conclusions

In summary, we compared the photoresponse characteristics of two types of GaN MSM photodetectors with a step-graded Al_x_Ga_1−x_N buffer and conventional HT-GaN/LT-AlN buffer layer. We investigated the effects of a buffer layers on the photoresponse characteristics of GaN MSM UV photodetectors. The GaN photodetector with the step-graded Al_x_Ga_1−x_N buffer showed a significantly enhanced photoresponsivity near the 365-nm wavelength range (UV-A region).
